# Catheter Ablation vs. Medical Treatment in Patients With Atrial Fibrillation

**DOI:** 10.7759/cureus.9700

**Published:** 2020-08-12

**Authors:** Suhail M Saad-Omer, Robert Ryad, Therese Limbana, Tehrim Zahid, Nusrat Jahan

**Affiliations:** 1 Medicine, California Institute of Behavioral Neurosciences & Psychology, Fairfield, USA; 2 Internal Medicine, California Institute of Behavioral Neurosciences & Psychology, Fairfield, USA; 3 Psychiatry, California Institute of Behavioral Neurosciences & Psychology, Fairfield, USA

**Keywords:** catheter ablation, medical treatment, atrial fibrillation

## Abstract

Atrial fibrillation has become the most commonly seen cardiac arrhythmia in clinical practice affecting almost 5.6 million Americans with that number expected to rise in the near future. The current literature review is aimed to assess the efficacy of catheter ablation in the treatment of patients with atrial fibrillation when compared to standard medical therapy. A PubMed search for studies of "Atrial Fibrillation" found 83,251 articles. Following the application of inclusion/exclusion criteria, we identified 44 articles of relevance that compared catheter ablation and medical therapy in the treatment of atrial fibrillation. These 44 articles included 20 Observational studies, eight randomized clinical trials, three clinical trials, five cohort studies, and eight review articles. Our review determined that catheter ablation was associated with a much lower rate of reoccurrence of atrial fibrillation when compared to medical therapy, as well as decreased cardiovascular outpatient visits and thromboembolic complications. The effect of quality on life when compared to medical treatment, however, was found to be inconclusive.

## Introduction and background

In recent years, atrial fibrillation (AF) has become the most commonly seen cardiac arrhythmia in clinical practice affecting an estimated 5.6 million Americans [1]. Due to an aging population and the increase of cardiac comorbidities, its prevalence is projected to rise to approximately 12 million Americans by 2050 [2]. This poses a major public health issue as AF patients have been shown to have increased hospitalizations due to stroke and heart failure, an increase in all-cause mortality, as well as considerably impaired quality of life (QOL) [3-4]. Altogether, this will have drastic effects on our health care system from both a resource and financial aspect [5].

The current approach for managing episodes of AF focuses on control of heart rate, prevention of thrombotic events and restoration and maintenance of sinus rhythm [6]. This is first done using medical management, which consists of using anti-arrhythmic agents like class 1A, 1C, 3A agents, and anticoagulants [7]. Despite being widely used, medical management has had debatable results with class 1 and 3A agents only being able to terminate about 50% of AF episodes, while amiodarone, a class 3 agent, only 70% [8]. They were all also associated with many side effects, including being proarrhythmic [9]. In addition to this, according to some studies, anti-arrhythmic medication treatment is also associated with the need for recurrent hospitalization in some patients [10]. This all resulted in the need to find alternative lines of treatment like catheter ablation (CA). CA was first introduced as a therapeutic option by Haïssaguerre et al. in 1998 [11]. They found that by electronically isolating the pulmonary veins, which were theorized to be the origin of ectopic beats that caused AF, they were able to terminate AF episodes and return them to normal sinus rhythm [12]. Thanks to significant technological advancement in recent years, this method of treatment has been found to be very effective.

Multiple randomized control trials (RCTs) have shown CA to be safer and superior to medical therapy in maintaining sinus rhythm and preventing reoccurrence of AF [13]. Similarly, many RCTs have consistently demonstrated an improvement in left ventricular ejection fraction (LVEF), QOL, and cardiovascular (CV) hospitalization with CA as compared with medical therapy [14]. Despite this, however, current professional guidelines recommend medical treatment as the initial line of management, whereas CA is usually reserved for symptomatic drug-refractory AF [9]. Due to the new advancements in the technology of CA and the addition of more evidence recently made available in the field [15], it is essential to review this information to determine the best method of treatment of patients suffering from AF. For this reason, this paper hopes to compare the use of medical treatment and catheter ablation in AF patients in relation to their various outcomes, including the ability to terminate episodes of AF, prevent AF reoccurrence, and the subsequent complications associated with either form of treatment. This will hopefully help to paint a clearer picture of the efficacy of CA in the treatment of AF and help provide more evidence in support of it becoming the new first-line measure of treatment.

## Review

Method

Literature was searched on PubMed using both Medical Subject Headings (MeSH) subheadings and regular keywords to collect data. Table 1 and Table 2 show regular and MeSH keywords search results.

**Table 1:** Regular keywords search results

Regular Keyword	Atrial Fibrillation	Catheter ablation vs. anti-arrhythmic agents in Atrial Fibrillation
Total articles	83,251	1,443
Articles selected	3,501	107

**Table 2:** MeSH keywords search results

MeSH keywords	Catheter Ablation + Anti-arrhythmia agents + Atrial Fibrillation
Total articles	1,155
Articles selected	141

Articles were chosen after applying the following inclusion/exclusion criteria.

Inclusion criteria:

1. Patients diagnosed with atrial fibrillation

2. Patients treated with either anti-arrhythmia agents, catheter ablation, or a combination of both

3. Paper written in English language and within the past six years

4. Study type is observational study, clinical trial, including randomized controlled trial, cohort study, case-control study, or review article

5. Full paper

Exclusion criteria:

1. Animal studies

2. Studies not written in the English language

3. Case report, case series, and meta-analysis

Results

Table 3 and Table 4 show regular and MeSH keyword search results after the application of the inclusion/exclusion criteria.

**Table 3:** Regular keywords search results after the application of inclusion/exclusion criteria

Regular Keyword	Atrial Fibrillation	Catheter ablation vs. anti-arrhythmic agents in Atrial Fibrillation
Total Articles	83,251	1,443
Inclusion/Exclusion		
Humans	68,223	1,392
English	58,994	1,203
Published within five years	18,501	333
Full text	3,501	107

**Table 4:** MeSH keywords search results after the application of inclusion/exclusion criteria

MeSH Keywords	Catheter Ablation + Anti-arrhythmia agents + Atrial Fibrillation
Total Articles	1,155
Inclusion/Exclusion	
Humans	1,152
English	1,002
Published within five years	283
Full text	141

3,394 articles from the keyword search “Atrial Fibrillation” were excluded due to a lack of focus on the management of this condition as well as the existence of duplicate articles. After a more thorough search, the total number of articles found was 107 full articles. All 107 were reviewed, and 63 were omitted because of one of the following reasons:

1. Study was focused on only AF patients with other underlying diseases like COPD or obesity

2. Included patients with arrhythmias other than AF

3. Case report or case series studies (as they only focus on one specific patient)

4. Meta-analysis

Finally, 44 publications in PubMed (with free full text available online) were reviewed, which included:

- 20 observational studies

- 11 random clinical trials, and three that were classified as clinical trials

- Five cohort studies

- Eight review articles

**Figure 1:**
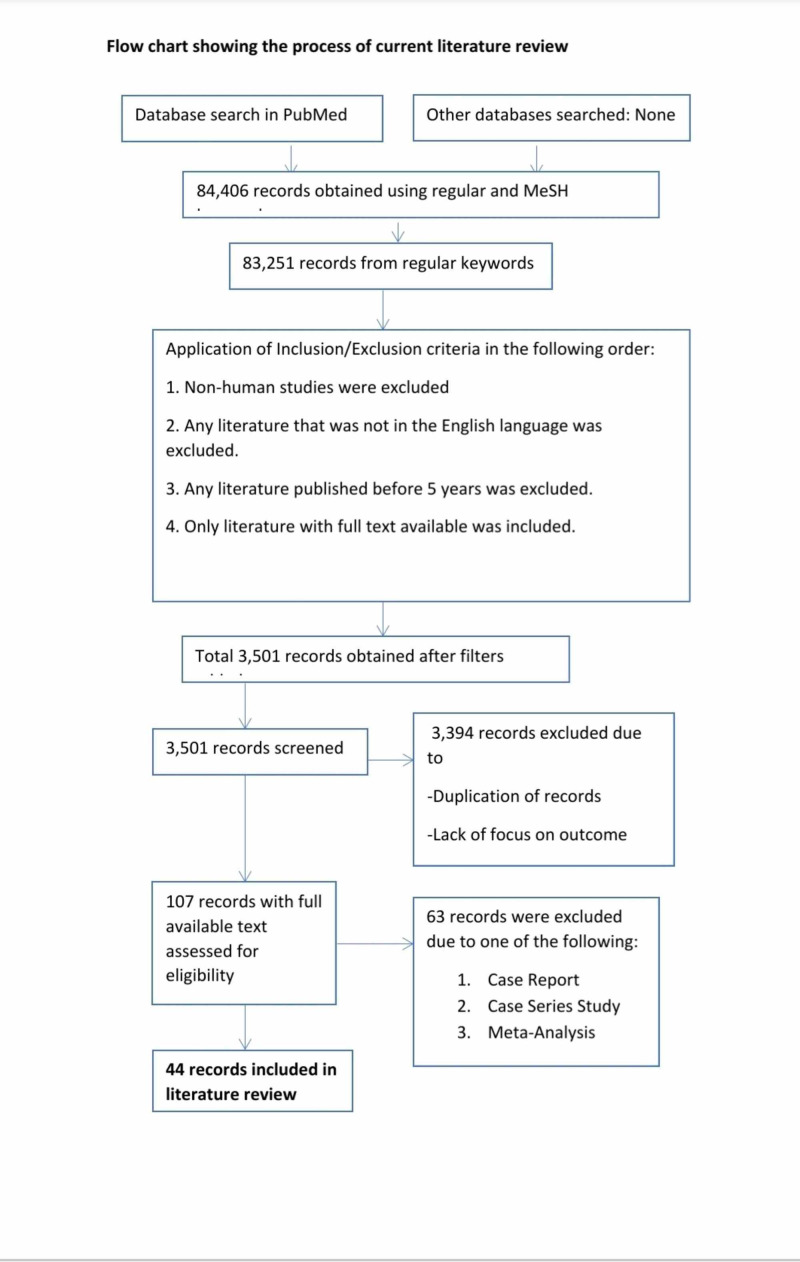
Flow chart showing the process of the current literature review

Figure 1 shows the flowchart illustrating the process of the current literature review.

Discussion

In this literature review, we examined the efficacy of using catheter ablation in treating patients diagnosed with atrial fibrillation when compared to standard medical therapy. We found that CA is associated with a significant reduction in AF recurrence. This finding was supported by multiple random clinical trials and observational studies as shown in Table 5 and Table 6. One RCT, the SARA study, showed that in 141 patients diagnosed with persistent AF, 70.8% of those who had CA done were free of any sustained episodes of AF at 12 months, while the same was true for only 43.7% in the anti-arrhythmic drug treatment (ADT) group [16]. Another RCT, RAAFT-2, showed similar findings in paroxysmal AF patients, with 54.5% of CA patients and 72.1% of ADT patients experiencing AF reoccurrences at two years [15]. This study does, however, reveal that despite the apparent benefits of CA, the reoccurrence rate is still high in both groups of patients. This sheds light on the fact that unfortunately, CA is not a one-off curative procedure for AF, with about 13.6% of patients in the CA group requiring a single additional ablation procedure or additional anti-arrhythmic therapy before they were able to return to sinus rhythm during the reported follow-up period. This is hypothesized to be due to the reconnection of one or more pulmonary veins, which are thought to be the source of the ectopic beats resulting in AF.

**Table 5:** Summary of RCTs comparing CA vs. Medical Therapy for AF treatment QOL: quality of life; CA: catheter ablation; AF: atrial fibrillation; ADT: anti-arrhythmic drug treatment; BMT: best medical therapy; AMICA: atrial fibrillation management in congestive heart failure with ablation; CNS: central nervous system; TIA: transient ischemic attack; MAFSI: Mayo AF-Specific Symptom Inventory; AAD: anti-arrhythmic drug

Author	Title	Study Design	Sample Size	Main Points
Blomström-Lundqvist et al. 2019 [17].	CAPTAF	RCT	155	Significant improvement in QOL of CA patients compared to ADT patients; decrease in AF burden in CA group compared to ADT group
Raatikainen et al. 2015 [18].	MANTRA-PAF	RCT	294	CA patients had significant difference of AF burden at 24 months; all groups showed a significant improvement in QOL; no evidence to suggest CA and medical therapy patients had any difference in adverse effects
Mont et al. 2015 [16].	SARA	RCT	146	70.4% in CA group and 43.7% in ADT group were free of any episode of AF at 12 months; cardioversion was less frequent in CA group (34.7% vs 50%)
Bertaglia et al. 2017 [19].	12-year follow-up of CA for AF: a prospective multicenter randomized Trial	RCT	137	27.9% of CA group vs 4.3% of ADT group had no relapse of atrial tachyarrhythmia; this was shown to be true with both paroxysmal and persistent AF
Kuck et al. 2019 [20].	AMICA	RCT	140	Sinus rhythm was recorded on 12-lead electrocardiograms at one year in 61/83 ablation patients (73.5%) and 42/84 BMT patients (50%); device-recorded AF burden at one year was 0% or maximally 5% of the time in 28/39 ablation patients (72%) and 16/36 BMT patients (44%); AMICA did not reveal any benefit of catheter ablation in patients with AF and advanced HF
Holmqvit et al. 2015 [21].	ORBIT-AF	CT	9,935	Only 5% of AF patients were previously treated with CA; lower proportion of minorities were treated with CA; no difference in stroke/ non-CNS TIA or death between CA and ADT groups
Marke et al. 2018 [22].	CABANA	RCT	2,204	CA patients had higher AFEQT scores compared to ADT patients (86.4 vs 80.9 respectively); the MAFSI frequency score showed better QOL for the catheter ablation group than the drug therapy group at 12 months (6.4 points vs 8.1 points)
Morillo et al. 2015 [15].	RAAFT-2	RCT	127	Atrial tachyarrhythmias > 30s were found in 72.1% vs 54.5% of ADT and CA patients respectively; difference in QOL was not statistically significant between both groups
Kaitani et al. 2016 [23].	EAST-AF	RCT	2,038	Patients assigned to AAD after CA were found after 90 days of treatment to have much higher event-free rates from recurrent atrial tachyarrhythmias when compared with the control group (59.0 and 52.1%, respectively); after one year, however, rates from the primary endpoint between the groups showed no major difference (69.5 and 67.8%, respectively)
Duytschaever et al. 2018 [24].	POWDER-AF	RCT	2,038	2.7% patients in the ADT-ON group vs. 21.9% patients in the ADT-OFF group were documented to have any atrial tachyarrhythmia lasting >30s at 12 months follow-up; repeat ablation was required less often in ADT-ON group (1.4% vs. 19.2%); both groups had corresponding QOL scores.

**Table 6:** Summary of observational studies comparing CA with medical therapy in the treatment of AF LA: left atrial; ERAF: early recurrence of atrial fibrillation; RC: rate control; MACEs: major adverse cardiac events; SR: sinus rhythm; LRAF: late recurrence of atrial fibrillation; RFCA: radiofrequency catheter ablation; LAD: left atrial diameter; LIPV SID: left inferior pulmonary vein superior–inferior diameter; PV: pulmonary vein; CPVI: circumferential pulmonary vein isolation

Author	Title	Study Design	Sample Size	Main Points
Mesquita et al. 2018 [25].	Very long-term outcomes after single catheter ablation	Observational	253	57% relapsed over a median five-year follow-up; annual relapse rate of 10% per year female sex, non-paroxysmal AF and LA volume predict reoccurrence
Yubing et al. 2019 [26].	Long-term outcome of radiofrequency CA for persistent AF	Observational	92	Maintenance of sinus rhythm was 40.2% after a single procedure, 52.2% after mean 1.3+/-0.6 procedures AF duration and ERAF were predictors of AF reoccurrence after single procedure
Geng et al. 2017 [27].	CA vs Rate Control in patients with AF and Heart Failure	Observational	90	82.2% of CA patients got freedom from AF; all patients in RC group remained in AF; CA group had decreased MACEs when compared to RC group (13.3% vs 29.3%)
Wang et al. 2019 [28].	RFCA for paroxysmal atrial fibrillation: outcomes during a 3-year follow-up period	Observational	243	At a median follow-up of 37 months after a single procedure, 60.5% of patients maintained SR; at a median follow-up of 42 months after multiple procedures, 74.9% of patients maintained SR; statistically significant risk factors for LRAF after a single RFCA session were the LAD, LIPV SID, PV number variation, CPVI combined with additional ablation, and ERAF.
Scherr et al. 2015 [29].	Five-year outcome of persistent AF ablation	Observational	150	Arrhythmia-free survival rates after a single procedure were 35.3±3.9%, 28.0±3.7%, and 16.8±3.2% at 1, 2, and 5 years, respectively; arrhythmia-free survival rates after the last procedure (mean 2.1±1.0 procedures) were 89.7±2.5%, 79.8±3.4%, and 62.9±4.5%, at 1, 2, and 5 years, respectively; failure to terminate AF during the index procedure, LAD, continuous AF >18 months and structural heart disease predicted AF reoccurrence

An observational study was carried out to determine whether repeat ablation, along with the continuation of medical management or medical management alone was more effective in the treatment of persistent AF [30]. It found that the patient group that received a single repeat ablation procedure was associated with a significant reduction in arrhythmia recurrences compared to the group that were given routine medical therapy alone. The importance of dual therapy was also shown by results from the POWDER-AF trial, where 147 paroxysmal AF patients were divided after ablation into ADT-ON and ADT-OFF groups [24]. The ADT-ON group had a lower rate of any documented atrial tachyarrhythmia lasting >30s at 2.7% compared to 21.9% in ADT-OFF group. The ADT-ON group also had a significantly lower rate of repeat ablation. The importance of using anti-arrhythmic therapy right after ablation is thought to be due to the fact that ablation uncovers non-pulmonary vein AF triggers in the left atrium that can be treated by the mechanism of action of various antiarrhythmic medications.

This literature review, however, also found the effect on QOL of CA compared to medical therapy to be inconclusive. One RCT, the CABANA trial, found that in 2,204 symptomatic patients with AF, CA patients noticed clinically essential and significant improvement in their QOL at 12 months [30]. These findings were reported using mean Atrial Fibrillation Effect on Quality of Life (AFEQT) summary score (range, 0-100; 0 indicates complete disability and 100 indicates no disability) and the Mayo AF-Specific Symptom Inventory (MAFSI) frequency score (range, 0-40; 0 indicates no symptoms and 40 indicates the most severe symptoms). However, the RAAFT-2 study showed that QOL, which was assessed using the EQ-5D tool, was improved from baseline in both the CA and medical therapy groups and the difference between the two was not statistically significant [15]. These conflicting results could be explained by the fact that these studies used two different systems to assess QOL as well as differences in their respective sample sizes. Hopefully, future research can help to develop a universal scoring system to be used to evaluate the QOL in AF patients.

Another observational study showed that although there was no statistically significant difference in the average number all-cause hospitalization or cardiac-related hospitalizations, CA patients required fewer cardiology-related outpatient visits when compared to patients receiving medical therapy [31]. This is important as it shows CA requires less resource use and lowers overall costs for the healthcare system. Another study supported this information and showed medical therapy patients had significantly higher hospitalization rates with much more of these hospital stays being due to thromboembolic events, especially strokes [32].

Despite all the discovered benefits associated with CA, physicians must still tread carefully when selecting this treatment for their patients. CA is an interventional procedure and is not without risks. Although associated with a low percentage, ranging from 3%-5% according to various studies [33], complications can still occur. Common complications found in this literature review include cardiac tamponade, pulmonary vein stenosis, and femoral artery pseudoaneurysms, among others. This stresses the importance of only carrying out this procedure in well-equipped facilities under highly trained operators and assessing the benefits of the procedure on a patient-specific basis.

Thanks to the addition of new technology, newer forms of CA have been discovered. Therefore, more research needs to be done comparing these forms to help guide patients management.

The current literature review has some limitations: no gender-specific analysis was performed, no particular age range determined, only studies published in the last six years were included, and no meta-analyses were included.

## Conclusions

The objective of our study was to review the efficacy of CA in the treatment of patients with AF when compared to standard medical treatment. The current literature review determined that patients treated with CA were found to have much lower rates of reoccurrence of AF, less risk of thromboembolic complications such as stroke, and decreased need for cardiology-related outpatient visits. CA was also associated with an improvement in QOL, but whether this is significantly more than that related to medical therapy remains inconclusive. This literature review adds more evidence in support of CA becoming the first-line treatment for AF. However, more research needs to be done in regard to the effect of CA on specific types of AF and to compare different methods of CA with each other. This will help determine the most effective modality of treatment and assist physicians in selecting the most beneficial management for their patients.
